# Smoking and Other Drug Characteristics of Aboriginal and Non-Aboriginal Prisoners in Australia

**DOI:** 10.1155/2013/516342

**Published:** 2013-03-31

**Authors:** Robyn L. Richmond, Devon Indig, Tony G. Butler, Kay A. Wilhelm, Vicki A. Archer, Alex D. Wodak

**Affiliations:** ^1^School of Public Health and Community Medicine, University of New South Wales, Kensington, Sydney, Australia; ^2^Centre for Health Research in Criminal Justice, Justice Health, University of New South Wales, Suite 302, Level 2, 152 Bunnerong Road, Pagewood, NSW 2035, Australia; ^3^National Centre in HIV Epidemiology and Clinical Research, University of New South Wales, Kensington, Sydney, Australia; ^4^School of Psychiatry, Faculty of Medicine, University of New South Wales, Faces in the Street, St. Vincent's Health Urban Mental Health Research, St Vincent's Hospital, Level 4, O'Brien Centre, St Vincent's Hospital, 390 Victoria Street, Sydney, NSW 2010, Australia; ^5^Alcohol and Drug Service, St. Vincent's Hospital, Darlinghurst, NSW 2010, Australia

## Abstract

*Introduction and Aim*. Although tobacco and alcohol use have declined substantially in the Australian community, substance use among prisoners remains high. The aim was to compare the smoking, drug, and alcohol characteristics, sociodemographic profile, and general health of Aboriginal and non-Aboriginal male prisoners in a smoking cessation intervention. *Design and Methods*. This study was a descriptive cross-sectional analysis of data from 425 male prisoners who joined a quit smoking trial conducted at 18 correctional centres in NSW and Queensland using data collected by standardised self-report instruments. *Results*. Average age was 33 years with 15% from Aboriginal descent. Compared to non-Aboriginal prisoners, Aboriginal prisoners were significantly more likely to have left school with no qualifications, to have been institutionalised as a child, to be previously incarcerated, and commenced smoking at a younger age. The tobacco use profile of both groups was similar; most of them had a medium to high level of nicotine dependence, smoked roll your own tobacco, and were “serious” about quitting. *Discussion and Conclusion*. Despite differences in terms of sociodemographic characteristics and offending history, the smoking characteristics of Aboriginal and non- Aboriginal prisoners were similar. Incarceration offers an opportunity to encourage smoking cessation and reduction of drug use.

## 1. Introduction

Tobacco use causes a higher burden of disease than other behavioural risk factors, contributing 9.5% of the total burden in men and 6% in women and causes 16,000 deaths in Australia each year [1]. The 2010 National Drug Strategy Household Survey reported that the daily smoking prevalence in Australia has declined from 22% in 2001 to around 15% in 2010 [2]. However, specific groups such as Aboriginal people had higher rates than non-Aboriginal Australians (38% versus 17%) [2]. This higher rate of smoking contributes to the substantially increased burden of illness and disease experienced by Aboriginal people and contributes to reduced quality of life and premature death [3–5]. The prevalence of smoking among prisoners in 2009 was over five times higher (76% versus 15%) than the general community, and Aboriginal male prisoners were more likely to smoke (83% versus 71%) than non-Aboriginal male prisoners [6, 7]. Smoking tobacco is socially acceptable and regarded as an integral part of prison life where it is used as a de facto currency, to relieve boredom and stress, and as a social lubricant [8, 9]. Despite these high rates of smoking among prisoners, 70% of currently smoking prisoners had attempted to quit smoking [7, 10].

Excessive alcohol consumption is also a major risk factor for ill health and death, causing 3.8% of the burden of disease for males and 0.7% for females [1] while illicit drug use contributes 2.0% to the burden [1]. Prisoner populations have high levels of drug use and the Inmate Health Survey reports 44% of women and 42% of men had used illicit drugs while in prison with cannabis and heroin being the most common [6, 11]. Drug use is a significant and multifaceted problem contributing to injury, social and family disruption, workplace concerns, violence, crime and community safety issues, morbidity, and mortality [12].

Aboriginal people are disproportionately represented among the Australian prisoner population, reflecting approximately 2% of the Australian population but 26% of the Australian prison population, with an incarceration rate 14 times higher than for non-Aboriginal people [13]. Since 1990, the proportion of Aboriginal men in prison in full-time custody in NSW has increased from 9.9% to 22.1% [14]. 

This paper consists of a detailed analysis of the baseline data for a larger randomised controlled trial for smoking cessation among male prisoners in NSW and Queensland. The purpose of this paper is to compare the sociodemographics, offending history, drug and alcohol use, and smoking behaviours of male prisoners, identifying any differences between Aboriginal and non-Aboriginal inmates. Measuring these characteristics is critical to understanding how best to develop appropriate and targeted interventions. Incarceration offers an important, but underutilised, public health opportunity to improve the health of several key disadvantaged population groups who are overrepresented among prisoners. 

## 2. Methods

From 2006 to 2010, we conducted a randomised controlled trial of a smoking cessation intervention among 425 male prisoners in 17 adult, men-only prisons in NSW and one prison in Queensland. Prisoners who were current smokers and who wanted to quit were invited to join the study through posters displayed in the prison clinics, flyers in the prisons, referrals from prison health clinic staff, and word of mouth between prison inmates. As a part of the study, we collected baseline data on demographic, smoking, and drug use characteristics.

### 2.1. Participants

Eligibility criteria for the intervention included male gender, current smoker, age over 18 years, no history of psychiatric illness (including current depression, as an antidepressant was used as an antismoking medication), no known allergy to nortriptyline or nicotine replacement therapy (NRT), no current treatment with another antidepressant or a major tranquiliser, and no history of cardiac disease (as determined by the medical records). Prisoners with current major depression were ineligible as they were likely to be taking antidepressant medication and there was potential for drug interactions, increased side effects and possibly increasing suicidality in a depressed prisoner. Female prisoners were not included as they only constitute 7% of the Australian prisoner population and tend to have much shorter sentences than men [13], thereby limiting availability for follow-up assessments at 6 and 12 months being conducted whilst in prison. 

### 2.2. Measures Used

Three prison nurse interviewers collected information in face-to-face interviews covering sociodemographic characteristics, offending history, smoking behaviour, prior attempts to quit smoking, physical health status, mental health (Kessler Psychological Distress Scale, K-10) [15], alcohol use before entering prison, and drug use before and during incarceration. The 12-item Short-Form Survey (SF-12) was used to assess overall physical and mental well-being using the Physical Component Summary and Mental Component Summary scores [16]. Blood pressure was measured at baseline, with high blood pressure defined as systolic blood pressure of 140 or more and/or diastolic blood pressure of 90 or more. “Sufficient physical activity” was defined as at least 150 minutes of walking, moderate or vigorous activity per week over at least 5 occasions (at least 600 minutes over a four-week period), based on a definition from the NSW Centre for Physical Activity and Health [17]. Participants completed the Fagerström Test for Nicotine Dependence [18] with a score of 6+ regarded as moderate to high nicotine dependence. We used the stages of change model which measures smokers' readiness to quit and has been well validated in general samples [19]. 

Carbon monoxide (CO) was measured as the biomarker for tobacco consumption and exposure, which provides an immediate objective measure of use with good correlation between breath test and self-reported smoking [20]. CO allows detection of smoking over a 6–24-hour period and is recognised as an appropriate measure of tobacco use [21]. In our study CO was measured at several assessment points, including baseline, in expired breath using the Micro 4 Smokerlyzer breath analyzer (Bedfont Scientific Ltd., UK) using the recommended cut point for smoking of 10 ppm [21]. 

### 2.3. Ethics Approvals

Prisoners provided written informed consent to join the study. The study was independently approved by the University of New South Wales (UNSW) Human Resources Ethics Committee, NSW Department of Corrective Services Ethics Committee, Justice Health Human Research and Ethics Committee, Aboriginal Health and Medical Research Council Ethics Committee, and Queensland Corrective Services Research Committee.

### 2.4. Data Analysis

Data analysis was conducted using SAS version 9.2 [22]. Descriptive statistics were used to compare the characteristics of Aboriginal and non-Aboriginal prisoners using chi-square and *t*-tests, with 0.05 level of significance.

## 3. Results

### 3.1. Demographics and Offending History

Of 1751 male inmates wishing to join our study, 1326 were ineligible. The main reasons for ineligibility were imminent release date (35%), history of psychiatric disease (40%), cardiac history (5%) and not yet sentenced (20%). The mean age of the 425 participants was 33 years (median 32.0, range 18 to 65 years), which was consistent with the median age of 33.6 of prisoners across Australia [13] and was similar among Aboriginal and non-Aboriginal prisoners (Table 1). In our sample, 15% were of Aboriginal descent (consistent with the 22% Aboriginal origin in NSW) [23] but lower than the 33% Aboriginal in the 2009 Inmate Health Survey that deliberately oversampled on Aboriginal people [6]. Demographic results from the participants in our study have been compared with the results from the Inmate Health Surveys to demonstrate the comparability of our population and thus the generalisability of our results.

All the following comparisons were made between Aboriginal and non-Aboriginal prisoners. Aboriginal prisoners were significantly more likely to have left school with no qualification (56% versus 41%, *P* < 0.03), a finding consistent with the 2009 Inmate Health Survey. Aboriginal prisoners also left school at a significantly younger age (14.5 years versus 15.3 years, *P* < 0.01). Significantly more Aboriginal prisoners (56% versus 35%, *P* < 0.01) reported being institutionalised as a child (i.e., juvenile detention and/or placed in care), consistent with 2009 Inmate Health Survey. Around three-quarters of prisoners (72%) were employed whilst in prison with no significant difference by Aboriginal status. 

A significantly higher proportion of Aboriginal prisoners had been previously incarcerated (77% versus 62%, *P* < 0.02) and had significantly more adult prison terms (a median of 3.0 versus 2.0, *P* < 0.02), both consistent with 2009 Inmate Health Survey. No other offending-related characteristics were significantly different by Aboriginality. Nearly a fifth (18%) of the sample had already been incarcerated for five or more years at the time of recruitment and over a third (34%) had a sentence length of five years or more.

Consistent with the 2009 Inmate Health Survey, there were no significant differences by Aboriginality for the SF-12 Physical or Mental Component Scores and the mean SF-12 Mental Component Score (44.1) was considerably lower than the community norm (standardised at 50.0) [16]. Also consistent with the 2009 Inmate Health Survey the level of physical activity was good with two thirds (68%) reporting sufficient exercise compared to one third (32%) of similarly aged males in the general population [24]. High blood pressure was rare for study participants (4.5%) and much lower than that found in the 2009 Inmate Health Survey (14.7%) [6].

### 3.2. Smoking and Cessation Behaviours

All participants were current smokers, a requirement for joining the study. By comparison, 75% of males from the 2009 Inmate Health Survey were current smokers, with significantly more Aboriginal men smoking than non-Aboriginal men (83% versus 71%, *P* < 0.01). Among smokers, the majority of smoking-related behaviours did not differ between Aboriginality, and non-Aboriginal prisoners, with the exception that Aboriginal prisoners started smoking at a significantly younger age (12.7 years versus 13.9 years, *P* < 0.05). In the 2009 Inmate Health Survey, Aboriginal men also started smoking at a younger age than non-Aboriginal men (13.6 years versus 14.0 years, *P* < 0.07), but this was not found to be statistically significant. Both Aboriginal and non-Aboriginal prisoners had smoked for an average of 20 years, having started daily smoking at 15.5 years (Table 2). The average number of cigarettes smoked daily was 23, with 70% of prisoners smoking 20 or more cigarettes per day. This was substantially higher than found in the 2009 Inmate Health Survey, where only 24% smoked 21 or more cigarettes per day and non-Aboriginal men were significantly more likely to smoke than Aboriginal men (27.5% versus 18.3%, *P* < 0.02) [6].

The most popular (97%) form of tobacco used was hand rolled cigarettes made from loose leaf “White Ox” tobacco. The majority (83%) of prisoners had medium to high levels of dependence (≥6 on Fagerstrom Test of Dependence) and the mean expired CO level was 14 ppm. Approximately one-third (34%) of the participants shared a cell with a smoker, a finding that was lower than found in the 2009 Inmate Health Survey. Significantly more Aboriginal prisoners indicated one of the main reasons they smoked was because their spouse or partner smoked or that it helped them to fit into groups (59% versus 37%, *P* < 0.01) compared with non-Aboriginal prisoners.

On average, prisoners reported trying to quit 2.6 times in the past, with 13% remaining abstinent for a month or more in the past year. Over half (54%) had successfully quit for one day in the past year. Significantly more Aboriginal prisoners had tried quitting in the past year by lowering tar or nicotine content of their tobacco (14% versus 5%, *P* < 0.01) than non-Aboriginal prisoners. The most popular methods for quitting while living in the community were going cold turkey, cutting down consumption, and using a nicotine patch.

Although all reported that they were serious about quitting and planned to stop smoking, only half (49%) were “very sure” they would be able to do so. There were no differences by Aboriginality regarding intention to quit smoking with most determined to quit and between 40%–50% very sure of their ability to do so. 

### 3.3. Drug and Alcohol Characteristics

Nearly all participants (95%) had “ever” used drugs but significantly more Aboriginal men had used drugs on a regular basis prior to prison (92% versus 79%, *P* < 0.02), consistent with the 2009 Inmate Health Survey. However, it is notable that the participants (all smokers) used drugs regularly at nearly twice the prevalence (81% versus 42%) as the general prison population in the 2009 Inmate Health Survey. This finding of increased drug use among smokers was consistent across all drug types and drug-related behaviours such as injecting and using drugs in prison. By contrast, risky alcohol use before entering prison was lower among the participants in our study who were all smokers compared to the general prisoner population in the 2009 Inmate Health Survey. Across both studies, Aboriginal men were significantly more likely than non-Aboriginal men to drink alcohol at risky levels prior to incarceration, including showing signs of risky and dependent drinking. 

Table 3 shows that, among Aboriginal men, regular use of cannabis was significantly higher (80% versus 57%, *P* < 0.01) than non-Aboriginal men and more than twice as high (61% versus 26%) as in the 2009 Inmate Health Survey. Regular use of all drugs was higher among participants in our study compared to the 2009 Inmate Health Survey participants, including heroin (37% versus 9%), other opiates (16% versus 4%), amphetamines (49% versus 14%), cocaine (36% versus 5%), and tranquilisers (17% versus 5%). There were no other significant differences in drug use characteristics by Aboriginality. Compared to non-Aboriginal men, in the 2009 Inmate Health Survey, Aboriginal men had significantly higher prevalence of regularly using heroin or other opiates and significantly higher prevalence of ever injecting heroin or amphetamines but lower prevalence (39% versus 48%, *P* < 0.02) of ever using cocaine [6]. 

## 4. Discussion

This is the first study that describes the sociodemographic and tobacco, drug, and alcohol use characteristics of a sample of male smoking prisoners by Aboriginality. 

People with low socioeconomic status, measured by level of education, are greatly overrepresented in prison. We found 44% of prisoners had left high school at Year 9 or before, with no educational qualification, a finding significantly higher for Aboriginal men. A similar result of 53% was reported in the 2009 NSW Inmate Health Survey, also significantly higher for Aboriginal men [7]. Smoking is related to lower education and lower income, two key variables of socio economic status [25–27]. Prisoner backgrounds are characterised by greater socioeconomic disadvantage as evidenced in lower levels of education, higher rates of unemployment prior to incarceration, and multiple contacts with the criminal justice system, with Aboriginal inmates experiencing greater disadvantage. Well-being has been found to be related to a high level of education [28]. However, in our study we report 17% of prisoners and as high as one quarter of Aboriginal prisoners rated their health as “fair” or “poor” in the last month.

Cardiovascular disease, diabetes, sexually transmitted infections, and ear and eye disorders and injury are in excess among Aboriginal people living in the community compared with the broad Australian population [5, 24]. Poor health among Aboriginal people is attributed to dispossession, forced separation of children from family and communities, and disadvantage [5]. Subjective well-being is positively related to education but negatively correlated to smoking, consistent with our findings of a generally low level of education and poor health among male smoking inmates. The finding that prisoners' health compares unfavourably with the general population has been reported in the surveys of prison inmates in NSW [14] and in other studies [29, 30]. We found that more than a third of our prisoner population had medical and mental health conditions, and most had cardiovascular risk factors [31]. Our findings are important as these health issues can be used to motivate smokers to quit their habit. These results lend credence to the view that the prisoner setting is an important context to deliver evidence based health promotion interventions to improve health outcomes and reduce morbidity and mortality.

The NSW Inmate Census of 2009 reports that tobacco use is over four times higher in the correctional system compared to the general population [2, 6]. This finding has also been reported in the US studies of prisoner populations [29, 30]. Smoking profiles were similar among Aboriginal and non-Aboriginal inmates except that Aboriginal inmates were found to have started smoking at a younger age. Previous research examining the physical and mental health of Aboriginal and non-Aboriginal inmates in NSW found little difference in health status between these two populations across a diverse range of health indicators [32, 33]. However, we report elsewhere that the cardiovascular health of prisoners is far worse than males of similar age from the most disadvantaged backgrounds in the general population [31]. In that study we also reported that Aboriginal prisoners had significantly more cardiovascular risk factors than non-Aboriginal prisoners [31]. 

We report that, among our sample of smokers, current regular drug use was high, particularly for Aboriginal men in prison. In our study we found that Aboriginal prisoners were significantly more likely than non-Aboriginal men to be regular cannabis users. Further, we found that regular cannabis use was more than twice as high as compared to the participants in the Inmate Health Survey (60% versus 26%) and six times higher than the 2010 National Drug Strategy Household Survey [2, 7]. From data gained from our focus group study [8] we found that drug use is common among inmates in prison, with many initiating drug use while incarcerated. The reason that the prevalence of tobacco, cannabis and drug use is higher in prison compared to the general population is that drug use in the correctional system is endemic with drugs including cannabis and tobacco used as currency by prisoners. 

Prisoners have a higher prevalence of illicit drug use compared to the broad Australian population [2, 6, 11]. Other research has identified strong associations between cannabis and tobacco among Aboriginal people, including the finding that cannabis use can sometimes be a gateway to tobacco use [34, 35]. Aboriginal men were also found to be significantly more likely than non-Aboriginal men to drink alcohol at risky or dependent levels prior to incarceration in both studies [34, 35]. There are 70% to 90% of heavy drinkers who are cigarette smokers [36, 37]. We expected that those who use tobacco as well as other drugs might find it more difficult to stop smoking compared with those who only smoked tobacco.

### 4.1. Strengths and Limitations of This Study

The strength of this study is that data are based on volunteer prisoners recruited to a smoking cessation RCT (to be reported elsewhere) who were representative of the general NSW prisoner population. Relying on self-reports may be imprecise and it is possible some respondents may have either minimised their tobacco use for social desirability or conversely exaggerated reports in order to be admitted to the randomised controlled trial to receive a smoking cessation intervention. This source of bias was minimised by the use of prison nurses as research assistants who have considerable experience taking medical and risk behaviour histories and working with the prisoner population. Further, we took biochemical measures (carbon monoxide) of tobacco use which provided an objective measure of smoking over a 6–24-hour period [21].

## 5. Conclusions

Only relatively recently have correctional communities realised the full extent of the burden of ill health which has been illuminated in the Inmate Health Surveys [6, 38]. However, these Inmate Health Surveys have limited information on tobacco and quit attempts. This paper provides unique information on tobacco use, quitting attempts and describes drug use in the prison setting. 

The correctional system offers public health opportunities to detect and treat diseases, educate on unsafe practices, promote health and behaviour change, and offer continuity of care. High rates of tobacco and other drug use among inmates, combined with an interest in quitting, are an ideal scenario to promote smoking cessation, reduced drug use and healthier lifestyles and to provide appropriate, evidence-based interventions. Proactive smoking cessation programs and drug modification interventions delivered in the corrections system to inmates who already have chronic health conditions and cardiovascular risk factors may have a direct health benefit on communities, when they are released, including significant cost savings and social benefits. We are encouraged by the large number of prisoners who were already engaged in physical activities to promote health and well-being and the number of those who want to stop smoking or cut down their consumption.

## Figures and Tables

**Table 1:** Demographics and offending history by Aboriginality.

Characteristics	Smoking cessation RCT			2009 NSW Inmate Health Survey		
	Aboriginal men % (*N* = 64)	Non-Aboriginal men % (*N* = 361)	Total men % (*N* = 425)	Aboriginal men % (*N* = 259)	Non-Aboriginal men % (*N* = 538)	Total men % (*N* = 797)
Mean age (±SD)	32.0 (9.7)	33.8 (10.2)	33.5 (10.2)	34.1^†^ (11.8)	36.1 (13.7)	35.5 (13.2)
Age group						
<25 years	28.1	22.2	23.1	37.1	33.5	34.6
25–29 years	23.4	19.4	20.0	8.9	10.6	10.0
30–39 years	21.9	29.9	28.7	18.5	17.0	17.5
40+ years	26.6	28.5	28.2	35.5	39.0	37.9
Aboriginal origin	—	—	15.1	—	—	32.5
Country of birth: Australia	100.0^†^	70.2	74.9	99.6^†^	72.7	81.4
Left school with no qualification	56.3^†^	41.3	43.5	72.5^†^	42.5	52.3
Mean age when left school (+SD)	14.5^†^ (1.7)	15.3 (2.0)	15.1 (2.0)	14.6 (1.3)	14.6 (1.2)	14.6 (1.2)
Institutionalised as a child (juvenile detention and/or placed in care)	56.3^†^	34.6	37.9	61.0^∗†^ 45.9^∧†^	32.9* 21.7^∧^	42.0* 29.6^∧^
Being homeless prior to prison	9.4	6.7	7.1	13.9	10.1	11.3
Employed while in prison	67.2	72.9	72.0	55.6	61.5	59.6
Offending history						
Previously incarcerated	76.6^†^	61.5	63.8	80.7^†^	55.9	64.0
Median number adult prison terms (+SD)	3.0^†^ (3.4)	2.0 (2.8)	2.0 (2.9)	3.0^†^ (3.8)	2.0 (3.0)	2.0 (3.3)
Incarcerated 5+ years at baseline	15.6	18.6	18.1	9.3	12.3	11.3
Sentence length 5+ years	35.9	33.8	34.1	29.8^†^	42.3	38.4
Health status						
SF-12: mean physical component score	51.8 (8.9)	52.4 (7.0)	52.3 (7.3)	51.7 (9.7)	52.0 (10.2)	51.9 (10.0)
SF-12: mean mental component score	43.2 (7.0)	44.2 (6.5)	44.1 (6.6)	44.6 (15.1)	44.3 (14.2)	44.4 (14.5)
Insufficient physical activity	23.4	22.7	22.8	20.7	23.9	22.8
High blood pressure (140+ sys or 90+ dias)	1.6	5.0	4.5	14.5	14.8	14.7
Fair/poor self-rated health	23.4	16.3	17.4	24.8	22.5	23.3

^†^Statistically significant *P* < 0.05 comparing Aboriginal and non-Aboriginal male inmates. N/A: not available. *Ever juvenile detention;  ^∧^ever placed in care as a child.

**Table 2:** Smoking and cessation behaviours by Aboriginality.

Characteristics	Smoking cessation RCT			2009 NSW inmate health survey		
	Aboriginal men % (*N* = 64)	Non-aboriginal men % (*N* = 361)	Total men % (*N* = 425)	Aboriginal men % (*N* = 259)	Non-aboriginal men % (*N* = 538)	Total men % (*N* = 797)
*Smoking behaviours and history *						
Current smoker	100.0	100.0	100.0	83.2^†^	71.1	
Mean age when first smoked tobacco (+SD)	12.7^†^ (4.3)	13.9 (4.2)	13.7 (4.3)	13.6 (5.0)	14.0 (4.5)	13.9 (4.7)
Mean age when first smoked tobacco daily (+SD)	15.1 (4.7)	15.5 (4.1)	15.5 (4.2)	N/A	N/A	N/A
Mean years when smoked tobacco (+SD)	19.2 (9.9)	19.9 (10.3)	19.8 (10.2)	N/A	N/A	N/A
Mean carbon monoxide reading (+SD)	13.1 (7.8)	14.5 (7.7)	14.3 (7.8)	N/A	N/A	N/A
Mean cigarettes smoked per day (+SD)	22.7 (8.4)	23.2 (10.0)	23.2 (9.8)	N/A	N/A	N/A
Smoking 20+ cigarettes per day	67.2	70.6	70.1	18.3^†∗^	27.5	24.2
High tobacco dependence (Fagerstrom 6+)	84.4	82.6	82.8	N/A	N/A	N/A
Sharing a cell with a smoker	42.2	31.9	33.5	62.9	51.9	55.1
Smoking White Ox (loose tobacco)	98.4	97.0	97.2	97.8	94.9	96.6
Reason for smoking: spouse smoked/helps fit into group	59.4^†^	36.6	40.0	N/A	N/A	N/A

*Smoking cessation history *						
Quitting behaviours in past year						
Giving up more than one month	12.5	13.0	12.9	17.2	22.1	20.4
Trying to give up unsuccessfully	65.6	59.3	60.2	43.7	52.4	49.3
Lower tar or nicotine content	14.1^†^	5.3	6.6	4.0	9.6	7.6
Reduced amount of tobacco smoked	50.0	47.4	47.8	56.3	57.2	56.9
Quitting on purpose for 24 hours	59.4	52.6	53.7	N/A	N/A	N/A
Any type of quitting behaviour	75.0	73.1	73.4	82.7	81.6	82.0
Mean times of trying to quit smoking (+SD)	2.8 (3.3)	2.6 (7.4)	2.6 (7.9)	N/A	N/A	N/A
Stages of readiness to quit smoking						
Seriously thinking of cutting down	98.4	94.7	95.3	N/A	N/A	N/A
Seriously thinking about quitting	100.0	99.7	99.8	N/A	N/A	N/A
Plannig to quit smoking	100.0	99.2	99.3	N/A	N/A	N/A
Very sure of wanting to cut down smoking	92.2	89.2	89.7	N/A	N/A	N/A
Very sure of being able to cut down smoking	43.8	48.2	47.5	N/A	N/A	N/A
Very determined to cut down	81.3	83.9	83.5	N/A	N/A	N/A
Very sure of being able to quit smoking	40.6	50.1	48.7	N/A	N/A	N/A
Very determined to quit smoking	90.6	86.7	87.3	N/A	N/A	N/A

^†^Statistically significant *P* < 0.05 comparing Aboriginal and non-Aboriginal male inmates. *Categories started at 21+ cigarettes per day.

**Table 3:** Drug and alcohol characteristics by Aboriginality.

Drug and alcohol use	Smoking cessation RCT			2009 NSW inmate health survey		
	Aboriginal men % (*N* = 64)	Non-aboriginal men % (*N* = 361)	Total men % (*N* = 425)	Aboriginal men % (*N* = 259)	Non-aboriginal men % (*N* = 538)	Total men % (*N* = 797)
Ever use any drugs	96.9	95.0	95.3	88.3	84.2	85.5
Regularly* use any drugs in year before prison	92.2^†^	79.0	80.9	50.8^†^	38.0	42.1
Ever use any drugs in prison	78.1^†^	63.2	65.4	48.0^†^	39.3	42.1
Ever inject any drugs	60.9	56.0	56.7	46.1^†^	37.2	40.1
Mean age when first injected (+SD)	18.9 (7.7)	17.9 (4.6)	18.0 (5.2)	18.5 (4.2)	18.5 (4.2)	18.5 (4.2)
Last injection in prison	32.4	33.7	33.5	22.0	22.2	22.2
AUDIT score 8+ prior to prison (risky drinker)	68.8^†^	48.5	51.5	74.2^†^	57.0	62.6
AUDIT score 20+ prior to prison (dependent drinker)	31.3^†^	18.6	20.5	44.1^†^	29.9	34.5
Drank 10+ drinks on typical day prior to prison	39.1^†^	24.7	26.8	57.8^†^	41.2	46.6
Drank 6+ drinks daily/almost daily prior to prison	26.6	19.9	20.9	39.8^†^	27.1	31.2
Cannabis						
Ever use	93.8	92.5	92.7	87.9^†^	81.6	83.6
Regularly use in year before prison	79.7^†^	57.1	60.5	34.4^†^	22.6	26.4
Ever use in prison	75.0^†^	57.1	59.8	35.9	31.2	32.7
Heroin						
Ever use	57.8	56.0	56.2	43.4	36.3	38.6
Regularly use in year before prison	40.6	37.1	37.7	13.7^†^	6.0	8.5
Ever inject	46.9	41.8	42.6	34.0^†^	26.5	28.9
Ever use in prison	32.8	34.9	34.6	17.2	15.2	15.9
Other opiates						
Ever use	37.5	36.8	36.9	20.7	18.2	19.0
Regularly use in year before prison	18.8	15.5	16.0	7.0^†^	3.0	4.3
Ever inject	29.7	24.1	24.9	15.6	12.6	13.6
Ever use in prison	15.6	19.4	18.8	5.9	5.6	5.7
Amphetamines						
Ever use	76.6	77.0	76.9	60.2	56.2	57.5
Regularly use in year before prison	46.9	49.0	48.7	17.2	13.0	14.3
Ever inject	53.1	44.6	45.9	37.5^†^	26.1	29.8
Ever use in prison	26.6	28.8	28.5	10.2	9.8	9.9
Cocaine						
Ever use	56.3	58.7	58.4	38.7^†^	47.9	44.9
Regularly use in year before prison	39.1	35.7	36.2	5.1	5.5	5.3
Ever inject	35.9	29.9	30.8	21.1	15.8	17.5
Ever use in prison	20.3	18.6	18.8	5.1	6.4	6.0
Tranquilisers						
Ever use	32.8	40.4	39.3	22.7	24.6	24.0
Regularly use in year before prison	15.6	17.7	17.4	5.1	5.5	5.3
Ever inject	6.3	7.8	7.5	3.1	3.6	3.4
Ever use in prison	18.8	18.0	18.1	6.3	8.1	7.5

^†^Statistically significant *P* < 0.05 comparing aboriginal and non-aboriginal male inmates. *Daily/almost daily use drugs prior to prison.
